# Insights into the internal structures of nanogels using a versatile asymmetric-flow field-flow fractionation method†

**DOI:** 10.1039/d0na00314j

**Published:** 2020-08-18

**Authors:** Edyta Niezabitowska, Adam R. Town, Bassem Sabagh, Marissa D. Morales Moctezuma, Victoria R. Kearns, Sebastian G. Spain, Steve P. Rannard, Tom O. McDonald

**Affiliations:** Department of Chemistry & Materials Innovation Factory, University of Liverpool Oxford Street Liverpool L69 7ZD UK tomm@liv.ac.uk +44 (0)151 795 0524; Postnova Analytics UK Ltd Units 64-65, Malvern Hills Science Park Malvern Worcestershire WR14 3SZ UK; Department of Chemistry, University of Sheffield Sheffield S3 7HF UK; Department of Eye and Vision Science, University of Liverpool Liverpool L7 8TX UK

## Abstract

Poly(*N*-isopropylacrylamide) (pNIPAM) nanogels are a highly researched type of colloidal material. In this work, we establish a versatile asymmetric-flow field-flow fractionation (AF4) method that can provide high resolution particle sizing and also structural information on nanogel samples from 65–310 nm in hydrodynamic diameter and so different chemical compositions. To achieve this online multi-angle light scattering and dynamic light scattering detectors were used to provide measurement of the radius of gyration (*R*_g_) and hydrodynamic radius (*R*_h_) respectively. Two different eluents and a range of cross-flows were evaluated in order to provide effective fractionation and high recovery for the different nanogel samples. We found that using 0.1 M NaNO_3_ as the eluent and an initial cross-flow of 1 mL min^−1^ provided optimal separation conditions for all samples tested. Using this method, we analysed two types of samples, pNIPAM nanogels prepared by free radical dispersion polymerisation with increasing diameters and analysed poly(acrylic acid)-*b*-pNIPAM crosslinked nanogels prepared by reversible addition–fragmentation chain transfer dispersion polymerisation. We could determine that the differently sized free radical nanogels possessed differing internal structures; shape factors (*R*_g_/*R*_h_) ranged from 0.58–0.73 and revealed that the smallest nanogel had a homogeneous internal crosslinking density, while the larger nanogels had a more densely crosslinked core compared to the shell. The poly(acrylic acid)-*b*-pNIPAM crosslinked nanogels displayed clear core–shell structures due to all the crosslinking being contained in the core of the nanogel.

## Abstract 2

This work demonstrates a versatile asymmetric-flow field-flow fractionation method for characterising the sizes and internal structures of poly(*N*-isopropylacrylamide) nanogels.

## Graphical abstract

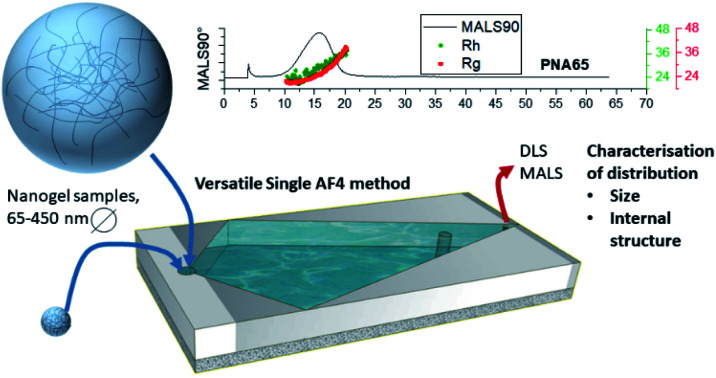

## Introduction

Nanogels and microgels are colloidal particles consisting of solvated, crosslinked polymeric networks. The terms nanogel and microgel are typically used interchangeably, and we will use nanogel from this point onwards. Nanogels composed of hydrophilic polymers have shown promise in a wide range of biomedical applications,^1^ but also in other applications such as enhanced oil recovery and sensing.^2^ Poly(*N*-isopropylacrylamide) (pNIPAM) based nanogels are a particularly well-known thermoresponsive nanogel. These particles undergo a deswelling transition upon heating to their volume phase transition temperature (VPTT) which is typically around 32 °C. The proximity of this temperature to human body temperature has been exploited for applications in drug delivery.^3^ For example, pNIPAM nanogels have been shown to provide triggered release of macromolecules^4^ and can produce long acting drug delivery implants upon injection into physiological environments.^5–7^ It has been shown that the internal structure of nanogels may be determined by their environment during formation; in particular, the formation of nanogels with core–shell type structures is highly dependent on synthesis conditions.^8^ Differences in the internal structure of nanogels may have a considerable impact on their properties,^7^ particularly in their use in *in situ* forming implants.^5,6^

In the field of nanoscience, it is important to obtain high resolution characterisation of particle sizes and information about internal structure. A promising approach to achieve this is to fractionate samples through the use of asymmetric field flow fractionation (AF4). AF4 offers reduced shear forces, avoids the need for stationary phases and can provide separations over a wide size range within the colloidal domain.^9^ These advantages have led to considerable growth in interest of field flow fractionation in fields such as nanomedicine.^10–15^ AF4 systems can be easily coupled to different detectors such as dynamic light scattering (DLS), multi-angle light scattering (MALS), UV-Vis spectrophotometry and inductively coupled plasma mass spectrometry (ICP-MS). These hyphenated approaches provide detailed information on a sample such as concentration of particles, size, molecular weight and shape for all particles within the sample.^16^

The use of AF4 for the analysis of aqueous nanodispersions is reasonably well established.^17^ However, despite the extensive amount of research undertaken on pNIPAM nanogels there are surprisingly few papers that have studied these materials by AF4. Smith *et al.* used AF4 to characterise the degradation of pNIPAM based nanogels with a hydrodynamic diameter of 132 nm.^18^ They used 3 mM NaN_3_ as the eluent with a two-step separation process. Firstly, a cross-flow of 1.0 mL min^−1^ was used which retained the nanogels while eluting degradation products. The nanogels were subsequently eluted in the second stage using a reduced cross-flow of 0.25 mL min^−1^. MALS was used for online characterisation with batch measurement of the hydrodynamic radius by DLS.^18^ Gaulding *et al.* used a 10 mM ionic strength aqueous buffer (containing NaNO_3_ and NaN_3_), and a variable cross-flow method from 1.0 mL min^−1^ to 0.1 mL min^−1^ to separate nanogels with hydrodynamic diameters of 96–146 nm from degraded polymer chains.^19^ In a later paper, Gaulding and coworkers used a 15 mM ionic strength pH 3.3 formate buffer to fractionate core–shell nanogels with hydrodynamic diameters of ∼260 nm. They achieved particle separation using a constant cross-flow of 0.25 mL min^−1^.^20^ All the samples analysed in these articles were pNIPAM based but the there were considerable differences between separation conditions used for analysis. This variability makes it difficult for researchers to select appropriate conditions for their pNIPAM nanogel samples.

AF4 also offers the opportunity to obtain information on the internal structure or the polymer architecture of a colloidal system.^21,22^ This understanding of size and morphology of particles is extremely valuable. Of particular interest for obtaining insight into the internal structure of samples is the combination of DLS and MALS.^21,23^ These techniques provide the hydrodynamic radius (*R*_h_) and the radius of gyration (*R*_g_) respectively. Information about the shape and conformation of particles can then be determined from the ratio *ρ* = *R*_g_/*R*_h_. This is a dimensionless value sometimes referred to as the shape factor.^24^ For particles, a shape factor of 0.78 indicates a sphere, ∼1 indicates vesicles, while values ∼2 reveals that the particles are potentially rods.^25,26^ Alternatively, soluble polymers in a random coil confirmation tend to give shape factor values in the range of 1.50–1.78.^27^ Thereby, determination of shape factor may give useful information about internal structure of nanogels. The shape factor of pNIPAM nanogels has previously been determined by using a combination of online and offline (batch) techniques. Static light scattering and DLS measurements below the VPTT have been used on nanogels with hydrodynamic diameters of 100–310 nm. This research has shown that increasing the crosslinking density caused a reduction in the shape factor from ∼0.9 to 0.6,^28,29^ while another article has shown that the crosslinking density had little impact on shape factor values 0.55–0.6.^27^ Small angle X-ray scattering (SAXS) has also been used to provide *R*_g_ measurement in the place of static light scattering.^8^ SAXS has also been used to investigate how the dispersion polymerisation synthesis conditions of pNIPAM influence the internal structure, showing that the larger nanogels tended to have more heterogeneous structures than smaller nanogels.^30^ It has been suggested that this heterogeneity is due to the different rates of monomer and crosslinker incorporation into the particles.^31^ The application of AF4 to the analysis of the internal structure of nanogels provides a number of potential benefits over the use of batch SAXS and SLS. Firstly, it provides high resolution fractionation of the particles which provides insight into the size distribution of a samples. Secondly, the use of the DLS and MALS detectors online gives a shape factor value for a sample much faster than a SAXS measurement.

In this work, we sought to develop a versatile AF4 method to characterise pNIPAM nanogel samples with *Z*-average hydrodynamic diameters from 65 to 310 nm and with different chemical/monomer compositions that would also provide insight into the internal structures of samples. Therefore, we evaluated separation conditions using different eluents and cross-flows with online MALS and DLS detectors for analysis. We demonstrate a method that can effectively fractionate different sized nanogel samples composed of pNIPAM and also poly(acrylic acid) and pNIPAM block copolymers. Additionally, this approach provides a clear insight into the internal structure of the nanogels.

## Experimental

### Materials

*N*-Isopropylacrylamide (NIPAM, ≥99%), *N*,*N*-methylenebis(acrylamide) (BIS, 99%), potassium persulfate (KPS, ≥99%), sodium chloride (NaCl, ≥99.5%), sodium nitrate (NaNO_3_, ≥99), sodium dodecyl sulfate (SDS, ≥99%), 4,4′-azobis(4-cyanovaleric acid) (ACVA, ≥98%) and 2,2′-azobis(2-methylpropionitrile) (AIBN) were purchased from Sigma-Aldrich Company Ltd, Gillingham (Dorset) UK, a subsidiary of Merck KGaA, Darmstadt, Germany. Acrylic acid (≥99%) was purchased from Merck. NIPAM was purchased from Fluorochem. Phosphate buffered saline tablets (PBS) were purchased from Fischer Scientific. Milli-Q water obtained from a water purification system had a resistivity of >18 MΩ cm^−1^ (PURELAB option R, Veolia). Spectra/por 2 (MWCO = 12–14 kDa) and spectra/por 3 (MWCO = 3.5 kDa) dialysis tubing was purchased from Spectrum Europe B.V., Breda, The Netherlands. Corning bottle top vacuum filter system with cellulose acetate membrane (pore size 0.22 μm) was purchased from Sigma-Aldrich Company Ltd, Gillingham (Dorset) UK, a subsidiary of Merck KGaA, Darmstadt, Germany. All reagents were used as supplied with the exception of the ACVA, NIPAM and AIBN that were used in the RAFT polymerisation as these were recrystallised from *n*-hexane and dried before use.

### Synthesis of pNIPAM nanogels by free radical dispersion polymerisation

The pNIPAM nanogels samples with four different mean diameters were synthesised by free radical dispersion polymerisation. The sample names are given as PNA#, with the number indicating the mean diameter in nm as measured by batch dynamic light scattering (DLS). The compositions used in the synthesis of each nanogel can be found in Table 1. The NIPAM monomer (7000 mg, 61.9 mmol), BIS crosslinker (700 mg, 4.5 mmol) and SDS surfactant (PNA310 = 78.8 mg, PNA160 = 260.2 mg, PNA100 = 701.6 mg PNA65 = 939.1 mg) were dissolved in deionised water (470 mL) in a 1 L two-neck round bottom flask equipped with a stir bar and reflux condenser. This was then sealed and nitrogen was bubbled through the aqueous solution for 1 hour whilst stirring (400 rpm) to remove dissolved oxygen. The solution was then heated to 70 °C. Separately KPS initiator (280 mg) was dissolved in distilled water (30 mL) and degassed with N_2_ for 1 hour before being transferred to the flask containing the monomers. The reaction was maintained under a N_2_ atmosphere for 4 hours at 70 °C before being cooled down to room temperature. The solution was then filtered through glass wool. To remove unreacted impurities, the nanogel suspension was dialyzed for 5 days using regenerated cellulose dialysis tubing (12–14 kDa MWCO for PNA310 and PNA160 and 3.5 kDa MWCO for PNA100 and PNA65), (Spectrum Labs), replacing the distilled water every 12 hours. The purified suspension was then lyophilized (Virtis Benchtop K with ultra-low temperature condenser) and stored in a desiccator.

**Table 1:** The composition used in nanogel synthesis

Sample	NIPAM (mg)	[SDS] (mg mL^−1^)	BIS (mg)	KPS^a^ (mg)	Water^b^ (ml)
PNA65	7000	1.88	700	280	500
PNA100	7000	1.40	700	280	500
PNA160	7000	0.52	700	280	500
PNA310	7000	0.16	700	280	500

a KPS dissolved at 9.3 mg mL^−1^ in distilled water.

b Total volume of water, including addition of KPS dissolved in water.

### Synthesis of poly(acrylic acid) macroCTA *via* ethanolic RAFT solution polymerisation

A mixture of 2-(hydroxyethylthiocarbonothioylthio)-2-methylpropanoic acid (0.56 g, 2.31 mmol), acrylic acid (10 g, 138.78 mmol), AIBN (0.076 g, 0.46 mmol) and ethanol to give a 25% w/w solids solution was purged thoroughly with N_2_ for 30 min. The flask was then placed onto a DrySyn® heating block preheated to 70 °C and left to react for 130 min. The reaction was quenched by removing the flask from the heat source and opening to air. Monomer conversion (78%) was determined by ^1^H NMR spectroscopy. The product was recovered by precipitation from diethyl ether (400 mL). Further product purification was performed by dialysis against water and freeze-drying to give a pale-yellow solid. *δ*_H_ (400 MHz; D_2_Ο, 25 °C) (ppm): 3.83 (2H, t, C*H*_2_OH), 3.60 (2H, t, C*H*_2_SC), 2.39 (1H, br. s, –C*H*–), 1.93–1.60 (2H, br. t, –C*H*_2_–); *δ*_C_ (100 MHz; D_2_O, 25 °C): 34.3 (–C*H*_2_–), 41.5 (–C*H*–), 178.9 (*C*(O)); *ν̄*_max_ (ATR) cm^−1^: 2935 (br. s, R–COOH), 1701 (vs, C=O), 1449 (w, –CH_2_–), 1411 (w, R–CH_2_–S), 1214 (m, –COOH), 1162 (s, C=S), 794 (m, –C–(CH_3_)_2_). Molecular weight determination of pAA by size exclusion chromatography was determined after esterification of the carboxylic groups. pAA samples were dissolved in THF/methanol mixes followed by the dropwise addition of trimethylsilyldiazomethane. Addition of the methylation agent ended when the production of N_2_ stopped, and the yellow colour remain unchanged. The solutions were left to stir allowing the solvents to evaporate overnight. The polymeric film was then dissolved in THF for GPC determination. *M*_n,theor_ = 3987 g mol^−1^; *M*_n,SEC_ = 4.7 kg mol^−1^, *M*_w_/*M*_n_ = 1.19.

### Synthesis of poly(acrylic acid)-*b*-poly(*N*-isopropylacrylamide) crosslinked nanogels *via* RAFT dispersion polymerisation

Two nanogel samples were prepared with different compositions with regards to the crosslinking density in the pNIPAM cores pAA_52_-*b*-p(NIPAM_200_-*co*-BIS_3_) or pAA_52_-*b*-p(NIPAM_200_-*co*-BIS_5_). pAA_52_ (0.0351 g, 0.009 mmol), NIPAM (0.2002 g, 1.770 mmol), BIS (0.0041 g, 0.027 mmol for 200 : 3 or 0.0069 g, 0.044 mmol for 200 : 5), were dissolved in water/EtOH (95/5 mole ratio) to give a 10% w/w solids solution. The pH of the solution was adjusted to pH 6.7 using a NaOH solution. The mixture was purged with N_2_ for 30 minutes, followed by the addition of ACVA in ethanol (0.120 mL, 14.27 mM). The mixture was then placed into an oil bath previously set at 70 °C and left to react for 18 h. Total monomer conversion (98% for 200 : 3 and 91% for 200 : 5) was estimated by moisture analysis. The product dispersion was purified by dialysis against DI water. *ν*/cm^−1^ (ATR-FTIR) 3290 (br. m, –CONH), 2973 (m, R–COOH), 2934 and 2873 (m, –CH_2_), 1640 and 1539 (s, –CONR_2_), 1457 (m, –CH_2_–), 1409 (w, –COOH), 1386 and 1367 (m, –C(CH_3_)_2_), 1172 (m, C=S), 1130 (m, –C(CH_3_)_2_), 879 (w, S=C(S)S).

### Characterisation of pNIPAM nanogels by AF4

**Fig. 1:**
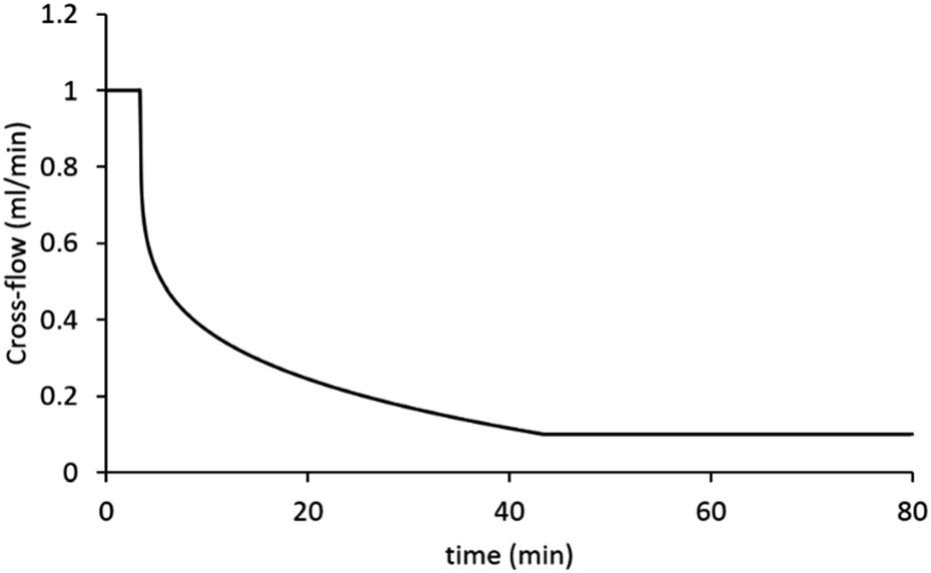
The cross-flow profile of the method chosen for separation of samples. The graph presents the change of cross-flow with time.

Asymmetric flow field flow fractionation (AF4) experiments were performed on an AF2000MT with RI and UV-Vis detectors from Postnova Analytics, Landsberg/Germany. A multi-angle light scattering detector (MALS) PN3621 from with a detector with 21 angles (from 7° to 164°) operating at 532 nm laser wavelength (set at 80% power) was coupled online to AF4. An autosampler (PN5300, Postnova) was used with the system. The hydrodynamic radii of the samples were obtained by DLS using a Malvern Zetasizer Nano ZS (running Malvern Zetasizer software V7.12) (Malvern Instruments, Malvern, UK) with 633 nm He–Ne laser and the detector positioned at 173°. The DLS measurements have been obtained using flow cell the Malvern quartz flow cell (ZEN0023) with flow rate 0.5 mL min^−1^ at 28 °C, coupled online to the AF2000MT. A 350 μm spacer and 10 kDa regenerated cellulose membrane were installed in the AF4 separation channel. The conditions used for the separations was based on a method existing in the literature.^19,32^ Briefly, the eluent was 0.1 M NaNO_3_ or 1× PBS (phosphate buffered saline) in Milli-Q H_2_O. Type I distilled water was obtained from a water purification system had a resistivity of >18 MΩ cm^−1^ (PURELAB option R, Veolia). The eluents were filtered using Corning bottle top vacuum filter system with cellulose acetate membrane with pore size 0.22 μm. The injected volume of the samples was 20 μL of a 4 mg mL^−1^ sample by autosampler. Each sample was analysed three times to check the reproducibility. A blank was measured between injections of new sample to ensure that there was no sample carry over. The UV-Vis detector measured two wavelengths 250 and 300 nm. The conditions used for the separations was as follows: the injection/focusing time was 3 min using a range of cross-flows from 2 to 0.5 mL min^−1^. The chosen cross-flow rate was kept constant for the first 0.2 minutes (*t*_0_–*t*_0.2_), and thereafter, the cross-flow was decreased in a power manner (exponent 0.2) from its initial value to 0.1 over a period of 40 minutes. Following the complete reduction in cross-flow, the tip-flow 0.4 mL min^−1^ continued for an additional 40 minutes. Except when specified otherwise, a constant detector flow rate of 0.5 mL min^−1^ was maintained at all times throughout injection, focusing and separation steps. The optimised method for fractionation conditions of the nanogels is shown in Fig. 1.

The recovery for pNIPAM samples was calculated using following equation:
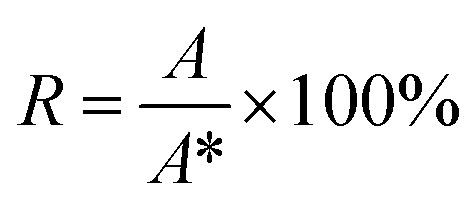
where *A* is the peak area of the nanoparticles with cross-flow, A* is the peak area without cross-flow, both obtained from the UV-Vis detector.^33^

### Dynamic light scattering in batch

Characterisation of the nanogels was carried out using dynamic light scattering (DLS) and laser Doppler electrophoresis (LDE). DLS and LDE were performed using a Malvern Zetasizer Nano ZS (running Malvern Zetasizer software V7.12) with 633 nm He–Ne laser and the detector positioned at 173°. Dialyzed samples were diluted to 1 mg mL^−1^. The *Z*-average diameter was recorded in the range (15–55 °C) using a thermal equilibration time of 600 seconds in 1 cm path length disposable polystyrene cuvettes. Measurements were repeated in triplicate to give a mean *Z*-average diameter and polydispersity index.

## Results and discussion

### Synthesis and characterisation of pNIPAM nanogels by free radical polymerisation

The pNIPAM nanogels were synthesised by free radical dispersion polymerisation in the presence of varying concentrations of SDS (0.16–1.88 mg mL^−1^) as the surfactant. This method of nanogel synthesis was first reported by Pelton *et al.*^34^ and, as reported in the literature, increasing the concentration of SDS reduced the size of particles. The resulting four different samples were analysed by DLS to obtain their hydrodynamic diameters in water at 25 °C. The DLS data showed narrow distributions with four hydrodynamic diameters spanning 60–315 nm and low polydispersity indices. The nanogels showed the usual thermoresponsive behaviour expected for pNIPAM nanogels; a deswelling transition at the volume phase transition temperature of 34 °C (see ESI, Fig. S1†). The four samples, PNA65, PNA100, PNA160 and PNA310 were named corresponding to their mean approximate hydrodynamic diameter at 25 °C. The hydrodynamic diameter and polydispersity index of the samples are shown in Table 2 and the monomodal particle size distributions from batch DLS are shown in ESI Fig. S2.†

**Table 2:** The four pNIPAM nanogel samples as measured by batch DLS at 25 °C

Sample	Hydrodynamic diameter (nm)	Polydispersity index
PNA65	63 ± 1.2	0.13 ± 0.008
PNA100	95 ± 0.8	0.14 ± 0.010
PNA160	165 ± 1.2	0.02 ± 0.012
PNA310	314 ± 4.0	0.01 ± 0.008

### Effect of eluent of fractionation behaviour

**Fig. 2:**
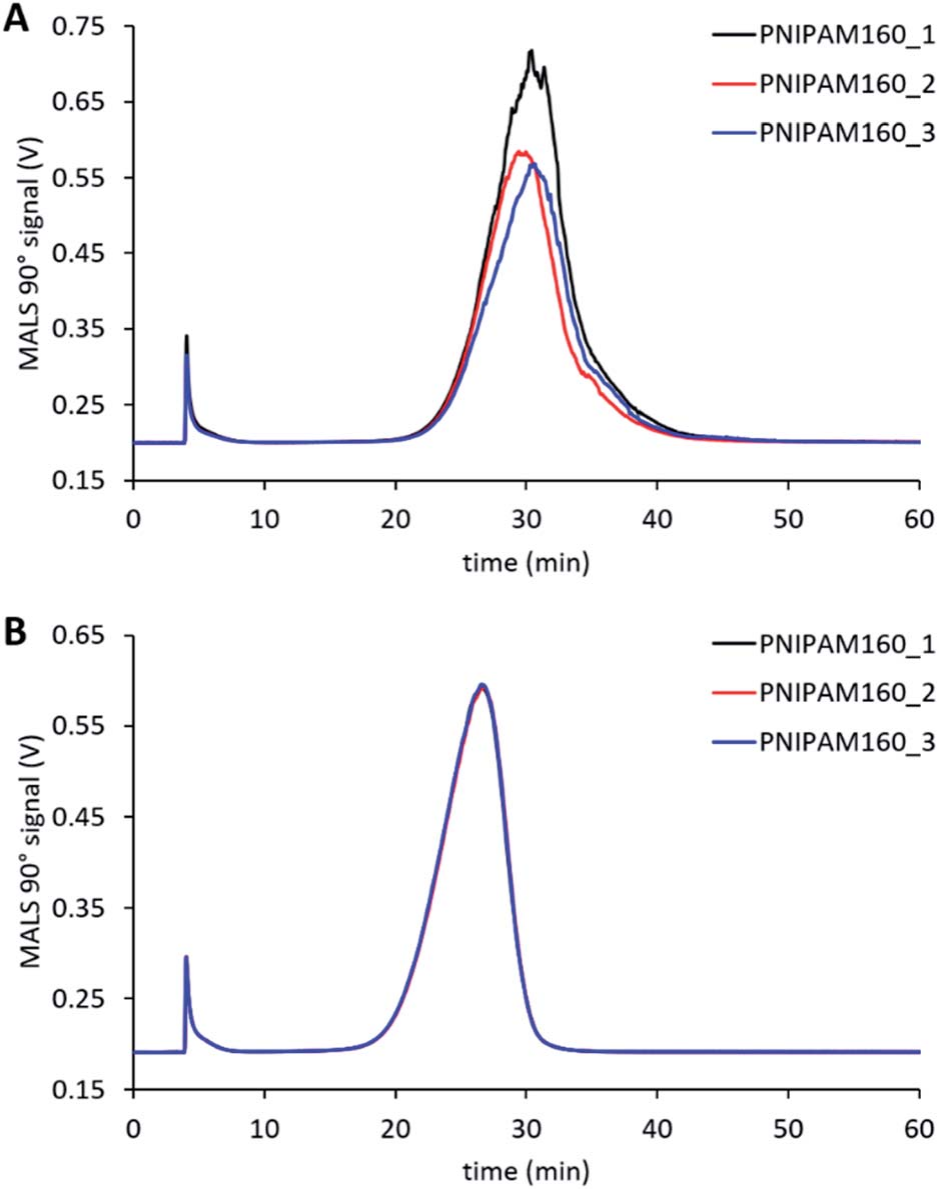
Comparison of the reproducibility using PBS (A) or NaNO_3_ (B) as the eluent for characterising PNA160 with an initial cross-flow of 1.0 mL min^−1^. The different data series are repeat runs of the same sample.

In order to evaluate the influence of different eluents previously used in the literature, both PBS^35,36^ and 0.1 M NaNO_3_ (ref. 19 and 37) were tested for fractionating nanogel samples. The reproducibility between three repeat injections in each eluent was checked (Fig. 2). These measurements showed considerably more variability between repeat injections for PBS compared to NaNO_3_. This difference may be attributed to increased interaction of the nanogels with the surface of membrane when dispersed in PBS. All the other nanogel samples also displayed highly reproducible separations in NaNO_3_ (see ESI Fig. S3†). Therefore, NaNO_3_ was selected as the eluent for further experiments. The thermoresponsive behaviour of pNIPAM means that the nanogels display a characteristic de-swelling upon heating above their volume phase transition temperature.^38^ Moreover, pNIPAM nanogels can also demonstrate dual-stimuli responsive behaviour where the combination of two stimuli such as temperature and ionic strength results in the nanogels losing colloidal stability and aggregating.^39^ The consideration of this behaviour is critical as aggregation must be avoided during AF4 analysis. Therefore, the colloidal stability of the nanogel samples was assessed by using batch DLS to monitor the change in hydrodynamic diameter in response to increasing temperature when dispersed in solution of 0.1 M NaNO_3_. All the nanogel samples showed a dramatic increase in the diameter at 34 °C, the volume phase transition temperature, (see ESI Fig. S4†) which revealed that the particles were aggregating. Therefore 28 °C was chosen as the temperature of separation for use in the AF4. This temperature was sufficiently below the VPTT to ensure that the nanogels were swollen and avoided the potential for aggregation. While also being sufficiently higher than room temperature to ensure that the multi-angle light scattering detector maintained a constant temperature. The choice of NaNO_3_ as the eluent and 28 °C yielded highly reproducible measurements and avoided potential issues caused by the thermoresponsive responsive properties of the nanogels.

### Effect of cross-flow rate

**Fig. 3:**
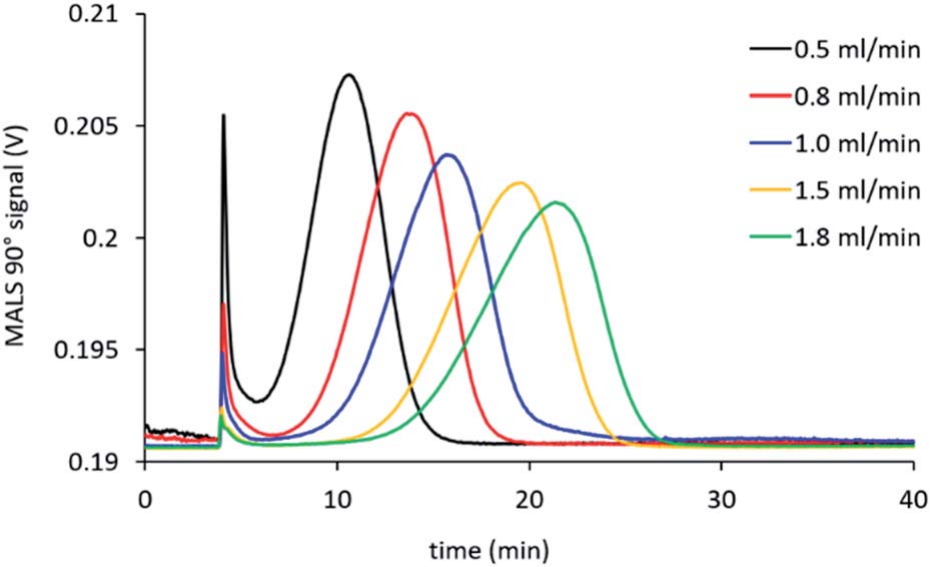
Effect of changing the initial cross-flow on the separation behaviour of PNA65 in NaNO_3_.

The cross-flow is one of the principal factors controlling retention time of particles and their effective separation in the AF4.^40^ Appropriate selection of this parameter needs to consider effective separation while avoiding adsorption of particles onto the membrane.^33^ For our method, we used a power reduction in the cross-flow (Fig. 1) as such methods combine effective fractionation with shorter run times. We therefore tested a range of initial cross-flows from between 0.5 mL min^−1^ to 2 mL min^−1^ for all four samples. Fig. 3 shows the separation behaviour of PNA65 at the different cross-flows as an example. Being the smallest in diameter, this sample would need the strongest separation force in order to fractionate the sample. The fractograms for the other nanogels are shown in ESI Fig. S5.† As expected, increasing the cross-flow increased the retention time for all samples. Additionally, increasing the cross-flow reduced the concentration of particles that were eluting in the void peak (seen at ∼5 minutes). Both of these behaviours were due to the increased separation force that is obtained by increasing the cross-flow.

As the objective of this work was to obtain a versatile single method to separate nanogel samples with a wide range of sizes, the use of a single cross-flow for all samples was desired. We, therefore, also determined the recovery at each cross-flow during separation of the samples. Typically, recovery can be obtained from UV-Vis absorbance, differential refractometer, fluorescence, or ICP-MS, where changes of analyte mass can be detected.^33^ We used an online UV-Vis detector to quantify the recovery and the calculated results are shown in Table 3.

**Table 3:** The calculated recovery for PNA65, PNA160, PNA310 based on area of peak obtained from UV-Vis detector with different initial cross-flow values

Crossflow (mL min^−1^)	Recovery (%)		
	PNA65	PNA160	PNA310
0.5	98	99	70
0.8	98	99	70
1	98	98	70
1.5	98	98	65
1.8	98	97	63
2	98	97	60

**Fig. 4:**
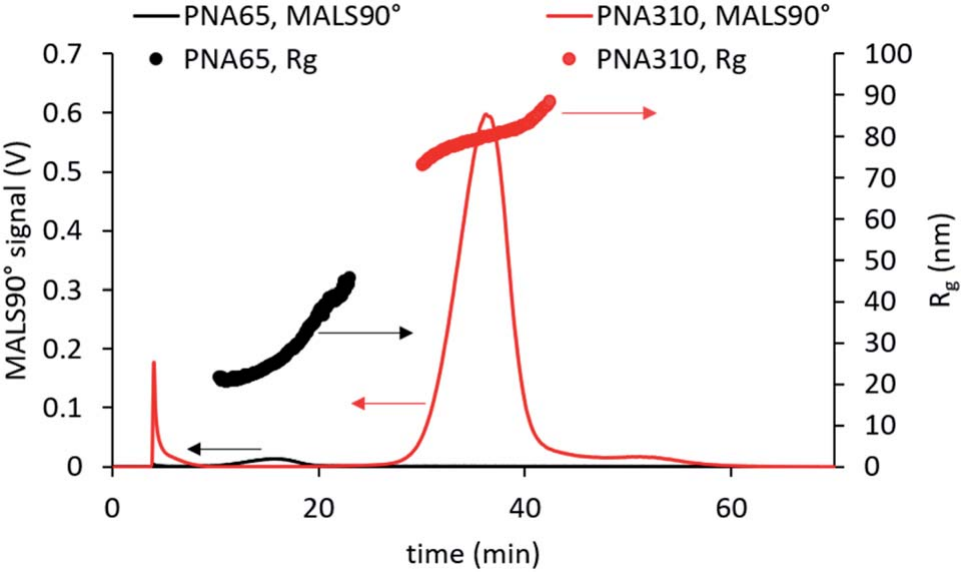
AF4-MALS analysis of the smallest and largest nanogels studied using an initial cross-flow of 1 mL min^−1^. This method led to effective fractionation with normal mode separation occurring for all samples.

Analysis of smaller nanogels (hydrodynamic diameter ≤ 160 nm) showed excellent recovery (*R* > 97%) for all tested cross-flows. However, for the nanogels with higher hydrodynamic diameter (>160 nm), the recovery decrease with higher cross-flows. This finding was due to the separation force being too strong for larger nanogels, which likely led to adsorption of the nanogels onto the membrane and lower recovery *R* < 65%. An initial cross-flow of 1 mL min^−1^ gave high recovery for all samples and displayed effective separation for all samples, as seen in the comparison of the fractograms for the largest and smallest nanogels in Fig. 4. Additionally, an increase in *R*_g_ with elution time was observed for each of the samples which indicates effective separation in normal mode.^33^

### Relationship between particle size and internal structure

**Fig. 5:**
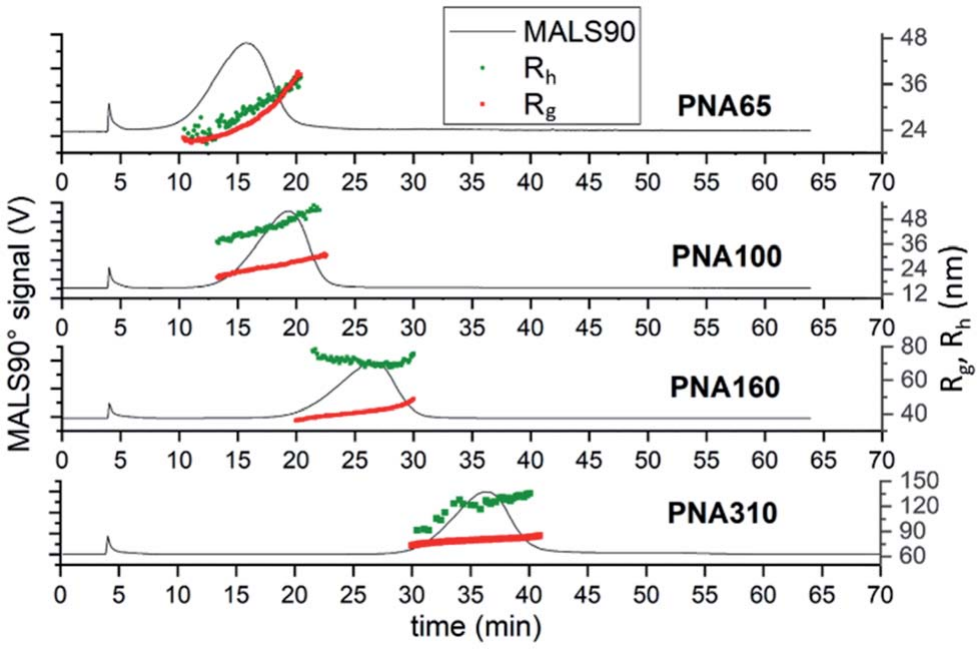
AF4-MALS-DLS analysis of nanogels. Fractograms showing the light 90° scattering detector signal (black line, left axis), radius of gyration (red series, right axis) and hydrodynamic radius (green series, right axis) in nanometers obtained from AF4-MALS-DLS measurements for PNA65, PNA100, PNA160 and PNA310.

Comparison of the fractograms for the four differently sized nanogel samples combined with the data obtained from MALS and DLS analysis (Fig. 5) provided insight into the structural differences between the samples. The signal obtained for 90° light scattering detector gives an indication of particle concentration and overlaid closely with the UV-Vis detector that measured actual concentration regardless of the nanogel size (ESI Fig. S6†). The online MALS and DLS detectors allow the measurements of *R*_g_ and *R*_h_ respectively, of the nanogels exiting the AF4 separation channel. The *R*_g_ values were calculated based on a fit for a spherical particle. The elution time for different nanogels can be seen to increase with increasing mean diameter; 16 min (PNA65), 19 min (PNA100), 26 min (PNA160) and 37 min (PNA310). Each nanogel sample also showed an increase in *R*_h_ with increasing elution time. AF4 separates based on the diffusion coefficients of the particles and therefore particles with a larger *R*_h_ are closer to the membrane and experience a reduced flow rate along the channel. Therefore the larger the *R*_h_, the longer the elution time.^15^ Some inconsistency was observed in the values for *R*_h_ obtained at either end of the size distribution, caused by the reduced signal-to-noise at the peak extremities due to the lower concentrations present. That said, the widths of the particle size distributions were relatively narrow for all the samples, with the difference between the smallest and largest particles in a population being less than 20 nm, except in the case of PNA310. For PNA310, the smallest particles eluting at 30 minutes were measured with a *R*_h_ ∼ 90 nm but this quickly increased to a *R*_h_ ∼ 120 nm at an elution time of 35 minutes. It is interesting to compare the widths of the distributions found by fractionation to the polydispersity index values obtained by batch DLS; the latter measurement indicated that the two smallest nanogel samples (PNA65 and PNA100) had the highest polydispersity index values (0.13 and 0.14) compared to the larger nanogels (PNA160, PNA310, at 0.02 and 0.01 respectively). This overestimation of the polydispersity index values by batch DLS was because the batch technique cannot resolve any additional scattering from larger contaminants in the nanogels. These larger contaminants, even if present at minute quantities, will scatter light much more intensely compared to the nanogels and therefore make the sample appear more polydisperse. Separation by AF4 before DLS measurements removes this issue and therefore gives a more accurate indication of the polydispersity. The *R*_g_ values for all samples also increased with increasing elution time as expected. For populations of nanoparticles with consistent internal structure *i.e.* a constant shape factor, the *R*_h_ and *R*_g_ should increase at the same rate. However, for a number of the nanogel samples this was not the case. For PNA65, the *R*_h_ displayed a constant gradient with elution time, but the *R*_g_ increases faster than expected, with the values for *R*_g_ becoming greater than *R*_h_ from elution times of ∼18 minutes. This indicated that the internal structure of this nanogel was changing with increasing size. A plot of the shape factors for all samples can be seen in ESI Fig. S7.† Such differences in the internal structure of the particles in the distribution might result from differences in the formation of the nanogels during the dispersion polymerisation.

The measurement of concentration along with the *R*_g_ measurement allowed the calculation of particle size distributions. Three of the four nanogel samples (PNA65, PNA100 and PNA160) had monomodal *R*_g_ size distributions that closely matched the mode MALS90 values taken from the fractograms (Fig. 5) which are given in Table 4. The exception was sample PNA310, which also had a second larger population of nanogels (containing ∼5% of the sample) with a mode of 135 nm (ESI Fig. S8†). Therefore, the values for the modes of the distributions can be considered to give an indication of the properties of the majority of the particles in the distribution (Table 4). The *R*_h_ values for the mode of the distribution generally agreed closely with the *R*_h_ obtained by the batch DLS measurement. The exception to this agreement was the difference between the online and batch values for the *R*_h_ for PNA310. It was previously been reported that by Sitar *et al.* that the size particles larger than approximately 100 nm may be underestimated by flow DLS. The authors suggested that the longitudinal flow velocity led to the DLS recording an overestimate in the diffusional movement.^41^ In order to investigate if this was the cause for the difference in our batch to flow *R*_h_ measurements, we analysed the effect of detector flow rate (0.5–0.2 mL min^−1^) on the size as measured by flow DLS (ESI Fig. S9†), if the longitudinal flow velocity was influencing the *R*_h_ values measured by DLS then slower flow rates would lead to larger *R*_h_ values being recorded. However, contrary to Sitar *et al.*^41^ we observed no effect of flow rate on the mode *R*_h_, indeed, we found that the lowest detect flow rate (0.2 mL min^−1^) reduced the efficiency of the fractionation of the sample. The reason for the difference in our findings and Sitar *et al.* is likely due to difference in the configuration of the flow cell in the DLS instruments used in our work and their work (unfortunately direct equipment comparison cannot be made as the flow cell was not reported by Sitar *et al.*) We believe that the difference between the batch and flow DLS measurements for PNA310 are instead due to the presence of a low quantity of secondary population of particles seen in Fig. 4 at elution times of 45–55 minutes, and also seen in the particle size distribution in ESI Fig. S8D.† These larger particles which will bias the batch DLS measurement to give a larger mean *R*_h_. The comparison of the shape factors for the differently sized nanogel samples reveal values ranging from 0.57–0.75 which are in agreement with literature values.^27,28,42^ The smallest nanogel possessed the largest shape factor with a value of 0.75 which is similar to that of a hard sphere with a constant internal polymer density (0.78), therefore indicating a close to homogeneous internal crosslinking density. The larger nanogels displayed lower values for the shape factor, ranging between 0.57–0.63. These values are typical for swollen nanogel with a denser inner core compared to the outer shell.^42^ All the nanogels were made at the same monomer composition but, the larger nanogels were obtained by using a lower concentration of the surfactant SDS in the dispersion polymerisation. It is difficult to obtain insight into the internal morphology of nanogels by electron microscopy, as the solvated polymer collapses upon drying. Indeed, we have previously shown that it was not possible to observe any difference in internal structure of the differently sized nanogels by scanning electron microscopy.^6^ However, it is clear that the internal structure of the nanogels has the potential to influence the packing behaviour of the nanogels; in our earlier work we found that PNA65 remained liquid at high concentrations (up to 24% w/v in water) while the larger nanogels with core–shell structures tended to form swollen gels due to volume blocking behaviour at concentrations greater than ∼7%.^7^ Our findings in this article, agree with what has previously been shown; that using larger amounts of SDS during the dispersion polymerisation route used for nanogels creates smaller more homogeneous particles,^30^ while using less SDS generates larger particles with a more heterogeneous structure which contains a dense gel particle core.^29^ Therefore, this AF4 separation method confirms the impact of the synthesis conditions on the internal structure of nanogels, and thus provides a clear insight into the internal structure of the nanogels.

**Table 4:** The mode values of *R*_g_, *R*_h_ and *ρ* (calculated from the AF4 flow measurements of *R*_g_/*R*_h_) for nanogels obtained from AF4-MALS-DLS for fractionation with 1 mL min^−1^ cross-flow^a^

Sample	*R*_g_ [nm]	*R*_h_ [nm]	*R*_h_ [nm] batch*	*ρ*
PNA65	25 ± 0.1	33 ± 1.0	31 ± 0.6	0.75
PNA100	26 ± 0.1	47 ± 0.3	43 ± 0.4	0.57
PNA160	42 ± 0.1	68 ± 1.0	75 ± 0.6	0.61
PNA310	80 ± 0.5	127 ± 0.3	180 ± 2.0	0.63

a *R*_h_ [nm] batch* have been measured by DLS using 1 mg mL^−1^ concentration of nanogels, at 28 °C in 0.1 M NaNO_3_.

### Synthesis and characterisation of copolymer nanogels prepared by controlled polymerisation

**Fig. 6:**
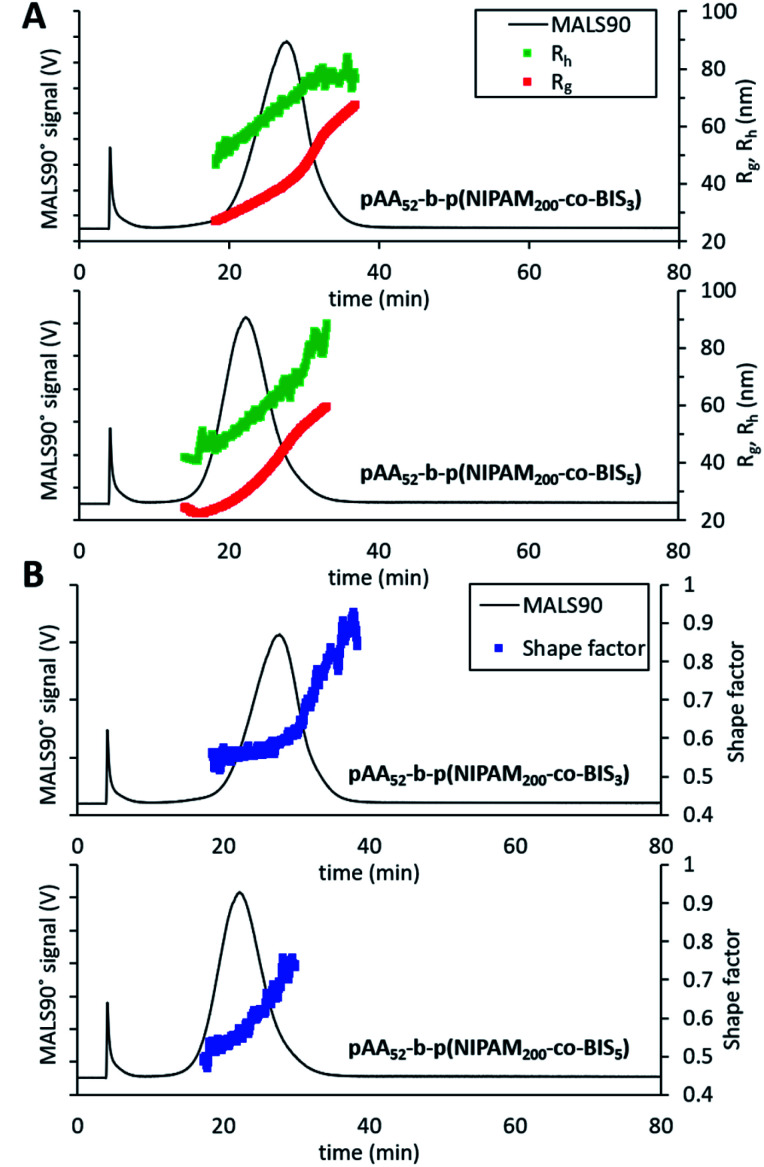
AF4-MALS-DLS analysis of RAFT nanogels. (A) Fractograms showing the light 90° scattering detector signal (black line, left axis), radius of gyration (red series, right axis) and hydrodynamic radius (green series, right axis) in nanometers obtained from AF4-MALS-DLS measurements for pAA_52_-*b*-p(NIPAM_200_-*co*-BIS_5_) and pAA_52_-*b*-p(NIPAM_200_-*co*-BIS_3_). (B) Shape factor for the two nanogel samples overlaid with the MALS 90° scattering detector signal (black line).

In order to further demonstrate the versatility of our method for characterising nanogels with different chemistries and structures we prepared copolymer nanogels of poly(acrylic acid) (pAA) and pNIPAM with well-defined core–shell structures. To achieve this, we used reversible addition–fragmentation chain transfer (RAFT) polymerisation to synthesise nanogels made by the chain extension of a pAA macro chain transfer agent (CTA) with degree of polymerisation (DP) of 52 with NIPAM and BIS as the crosslinker. The resulting nanogels therefore possessed a core of pNIPAM crosslinked by BIS with a shell of PAA. A DP of 200 was targeted for the pNIPAM core and two samples were prepared in which the crosslinking density of core was varied in terms of NIPAM : BIS molar ratios, to give two samples with the following compositions pAA_52_-*b*-p(NIPAM_200_-*co*-BIS_3_) or pAA_52_-*b*-p(NIPAM_200_-*co*-BIS_5_). The resulting samples were then analysed by the AF4 method we had developed for the earlier pNIPAM nanogels made by free radical polymerisation. Both samples displayed effective fractionation allowing measurement of the *R*_g_ and *R*_h_ values (Fig. 6A) and were monomodal as seen in the particle size distributions (ESI Fig. S10†).

The sample with the lower crosslinking density (pAA_52_-*b*-p(NIPAM_200_-*co*-BIS_3_)) was found to elute after a longer time period and the *R*_g_ and *R*_h_ measurements showed it was the larger of the two samples (mode values are shown in Table 5). This difference in the sizes of the particles was likely due to the looser crosslinking in the NIPAM core enabling greater swelling. Analysis of the shape factors (Fig. 6B) revealed more details about the internal structure of the nanogels. For pAA_52_-*b*-p(NIPAM_200_-*co*-BIS_3_), the sample displayed a gradual increase from 0.5 up to 0.55 for the majority of the particles in the distribution. After an elution time or 30.7 minutes, there was followed by a dramatic increase in the shape factor up to ∼0.90. This trend indicates that the larger particles in the population of possessed different internal structures compared to the majority of the sample. The smaller, more numerous population of particles appeared to possess core–shell structures in which the core is denser. While the larger and less numerous population likely possessed less well-defined structures. The pAA_52_-*b*-p(NIPAM_200_-*co*-BIS_5_) sample displayed a gradual increase in the shape factor values from 0.48 to 0.75. The use of controlled polymerisation to form these core–shell nanogels means that the thickness of the shell (controlled by the DP of the pAA macroCTA) was fixed for all particles in the distribution. Therefore, as the nanogels increased in diameter, a greater proportion of the particles were made of the pNIPAM crosslinked with BIS which will have possessed a higher density. This structural change in the nanogels explains the gradual increases in the shape factor values for both samples with increasing size. This analysis of the copolymer nanogels by AF4 further reinforces the value of our versatile method by enabling the analysis of nanogels with differing sizes and also different surface chemistries.

**Table 5:** The mode values of *R*_g_, *R*_h_ and *ρ* (calculated from the AF4 flow measurements of *R*_g_/*R*_h_) for nanogels obtained from AF4-MALS-DLS for fractionation with an initial 1 mL min^−1^ cross-flow

Sample	*R*_g_ [nm]	*R*_h_ [nm]	*ρ*
pAA_52_-*b*-p(NIPAM_200_-*co*-BIS_3_)	40 ± 0.5	69 ± 0.25	0.59
pAA_52_-*b*-p(NIPAM_200_-*co*-BIS_5_)	31 ± 0.54	55 ± 0.49	0.57

## Conclusions

This study has demonstrated the value of AF4 coupled online with MALS and DLS for analysing nanogel samples from 65–310 nm in diameter composed of both pNIPAM nanogels prepared by free radical polymerisation and pAA-*b*-pNIPAM core–shell nanogels prepared by RAFT polymerisation. The diameters of particles and information on the internal structure of the nanogels can be easily obtained by using a single AF4-MALS-DLS fractionation method. During these studies, we have successfully fractionated PNA nanogels obtaining radius of gyration, hydrodynamic radius and shape factors for all six samples. The chosen eluent method showed good reproducibility and high resolution of sizes for AF4-MALS-DLS measurements. The fractionation of the samples revealed that the internal structure of some of the samples varied within the distribution. Such information cannot be obtained through the use of bulk analysis methods such as SAXS. For the nanogels prepared by free-radical polymerisation, determination of the mode values of *R*_g_/*R*_h_ for the distribution of the nanogels showed that diameters higher than 100 nm had values ∼0.61, indicating that the particles showed more like core–shell structures. The analysis of the smallest nanogel (65 nm) gave *R*_g_/*R*_h_ values 0.78 indicating more homogenous structures. These measurements showed that the synthesis conditions have a significant impact on the internal structure of nanogels. For the core–shell pAA-*b*-pNIPAM nanogels, the separation effectively fractionated the nanogels and core–shell structure was clearly revealed. In the future, analysis of the fractal dimensions might provide further insight into internal structure of the nanogels.^43^ Our work will be of importance to researchers working with aqueous nanogels/microgels, by providing a versatile method for obtaining a clear insight into the internal structures of nanogels within differently sized populations of particles.

## Conflicts of interest

BS is an employee of Postnova Analytics, a manufacturer of AF4 instruments. The authors declare no other conflicting interests.

## Supplementary Material

NA-002-D0NA00314J-s001

## References

[cit1] Oh J. K., Drumright R., Siegwart D. J., Matyjaszewski K. (2008). Prog. Polym. Sci..

[cit2] Thorne J. B., Vine G. J., Snowden M. J. (2011). Colloid Polym. Sci..

[cit3] Ekkelenkamp A. E., Elzes M. R., Engbersen J. F. J., Paulusse J. M. J. (2017). J. Mater. Chem. B.

[cit4] Smeets N. M. B., Hoare T. (2013). J. Polym. Sci., Part A: Polym. Chem..

[cit5] Town A. R., Giardiello M., Gurjar R., Siccardi M., Briggs M. E., Akhtar R., McDonald T. O. (2017). Nanoscale.

[cit6] Town A. R., Taylor J., Dawson K., Niezabitowska E., Elbaz N. M., Corker A., Garcia-Tuñón E., McDonald T. O. (2019). J. Mater. Chem. B.

[cit7] Town A., Niezabitowska E., Kavanagh J., Barrow M., Kearns V. R., García-Tuñón E., McDonald T. O. (2019). J. Phys. Chem. B.

[cit8] Stieger M., Richtering W., Pedersen J. S., Lindner P. (2004). J. Chem. Phys..

[cit9] Messaud F. A., Sanderson R. D., Runyon J. R., Otte T., Pasch H., Williams S. K. R. (2009). Prog. Polym. Sci..

[cit10] Yohannes G., Jussila M., Hartonen K., Riekkola M. L. (2011). J. Chromatogr. A.

[cit11] Weber C., Simon J., Mailänder V., Morsbach S., Landfester K. (2018). Acta Biomater..

[cit12] Contado C. (2017). Anal. Bioanal. Chem..

[cit13] Iavicoli P., Urbán P., Bella A., Ryadnov M. G., Rossi F., Calzolai L. (2015). J. Chromatogr. A.

[cit14] Zattoni A., Rambaldi D. C., Reschiglian P., Melucci M., Krol S., Garcia A. M. C., Sanz-Medel A., Roessner D., Johann C. (2009). J. Chromatogr. A.

[cit15] Wagner M., Holzschuh S., Traeger A., Fahr A., Schubert U. S. (2014). Anal. Chem..

[cit16] Pasch H., Makan A. C., Chirowodza H., Ngaza N., Hiller W. (2014). Anal. Bioanal. Chem..

[cit17] Ratanathanawongs Williams S. K., Lee D. (2006). J. Sep. Sci..

[cit18] Smith M. H., South A. B., Gaulding J. C., Lyon L. A., Medeiros G. A., Bataglion G. A., Ferreira D. A. C., De Oliveira H. C. B., Eberlin M. N., Neto B. A. D., Chemistry T., De Brasilia U., Mass T. (2010). Anal. Chem..

[cit19] Gaulding J. C., Smith M. H., Hyatt J. S., Fernandez-Nieves A., Lyon L. A. (2012). Macromolecules.

[cit20] Gaulding J. C., South A. B., Lyon L. A. (2013). Colloid Polym. Sci..

[cit21] Boye S., Ennen F., Scharfenberg L., Appelhans D., Nilsson L., Lederer A. (2015). Macromolecules.

[cit22] Fuentes C., Castillo J., Vila J., Nilsson L. (2018). Anal. Bioanal. Chem..

[cit23] Vezočnik V., Rebolj K., Sitar S., Ota K., Tušek-Žnidarič M., Štrus J., Sepčić K., Pahovnik D., Maček P., Žagar E. (2015). J. Chromatogr. A.

[cit24] Burchard W. , in Light Scattering from Polymers, 2007, vol. 48, pp. 1–124

[cit25] Brewer A. K., Striegel A. M. (2009). Anal. Bioanal. Chem..

[cit26] Brewer A. K., Striegel A. M. (2011). Analyst.

[cit27] Senff H., Richtering W. (2000). Colloid Polym. Sci..

[cit28] Varga I., Gilányi T., Mészáros R., Filipcsei G., Zrínyi M. (2001). J. Phys. Chem. B.

[cit29] Arleth L., Xia X., Hjelm R. P., Wu J., Zhibinc H. U. (2005). J. Polym. Sci., Part B: Polym. Phys..

[cit30] Andersson M., Maunu S. L. (2006). J. Polym. Sci., Part B: Polym. Phys..

[cit31] Acciaro R., Gilanyi T., Varga I. (2011). Langmuir.

[cit32] Rebolj K., Pahovnik D., Žagar E. (2012). Anal. Chem..

[cit33] Gigault J., Pettibone J. M., Schmitt C., Hackley V. A. (2014). Anal. Chim. Acta.

[cit34] McPhee W., Tam K. C., Pelton R. (1993). J. Colloid Interface Sci..

[cit35] Maruyama A., Winnik F. M., Kano A., Moquin A., Akiyoshi K., Sawada S., Shimoda A. (2011). Colloids Surf. B Biointerfaces.

[cit36] Hong J. S., Stavis S. M., Depaoli Lacerda S. H., Locascio L. E., Raghavan S. R., Gaitan M. (2010). Langmuir.

[cit37] Hupfeld S., Ausbacher D., Brandl M. (2009). J. Sep. Sci..

[cit38] Muratalin M., Luckham P. F., Esimova A., Aidarova S., Mutaliyeva B., Madybekova G., Sharipova A., Issayeva A. (2017). Colloid. Surface. Physicochem. Eng. Aspect..

[cit39] Rasmusson M., Routh A., Vincent B. (2004). Langmuir.

[cit40] Omar J., Boix A., Kerckhove G., von Holst C. (2016). Food Addit. Contam., Part A.

[cit41] Sitar S., Vezočnik V., Maček P., Kogej K., Pahovnik D., Žagar E. (2017). Anal. Chem..

[cit42] Deen G. R., Pedersen J. S. (2015). Cogent Chem..

[cit43] Gennari A., de la Rosa J. M. R., Hohn E., Pelliccia M., Lallana E., Donno R., Tirella A., Tirelli N. (2019). Beilstein J. Nanotechnol..

