# Supplementary material for: Insights into the internal structures of nanogels using a versatile asymmetric-flow field-flow fractionation method
Source: Nanoscale Adv. 2020 Aug 18;2(10):4713–21. doi: 10.1039/d0na00314j (PMC9416902; doi:10.1039/d0na00314j)
Supplement: NA-002-D0NA00314J-s001 [file NA-002-D0NA00314J-s001.pdf]

Electronic Supplementary Material (ESI) for Nanoscale Advances.
This journal is © The Royal Society of Chemistry 2020

Supporting Information

Insights into the Internal Structures of Nanogels Using a Versatile Asymmetric-Flow Field-Flow Fractionation Method.

Edyta Niezabitowska, Adam R. Town, Bassem Sabagh, Marissa D. Morales Moctezuma, Victoria R. Kearns, Sebastian G. Spain, Steve P. Rannard and Tom O. McDonald

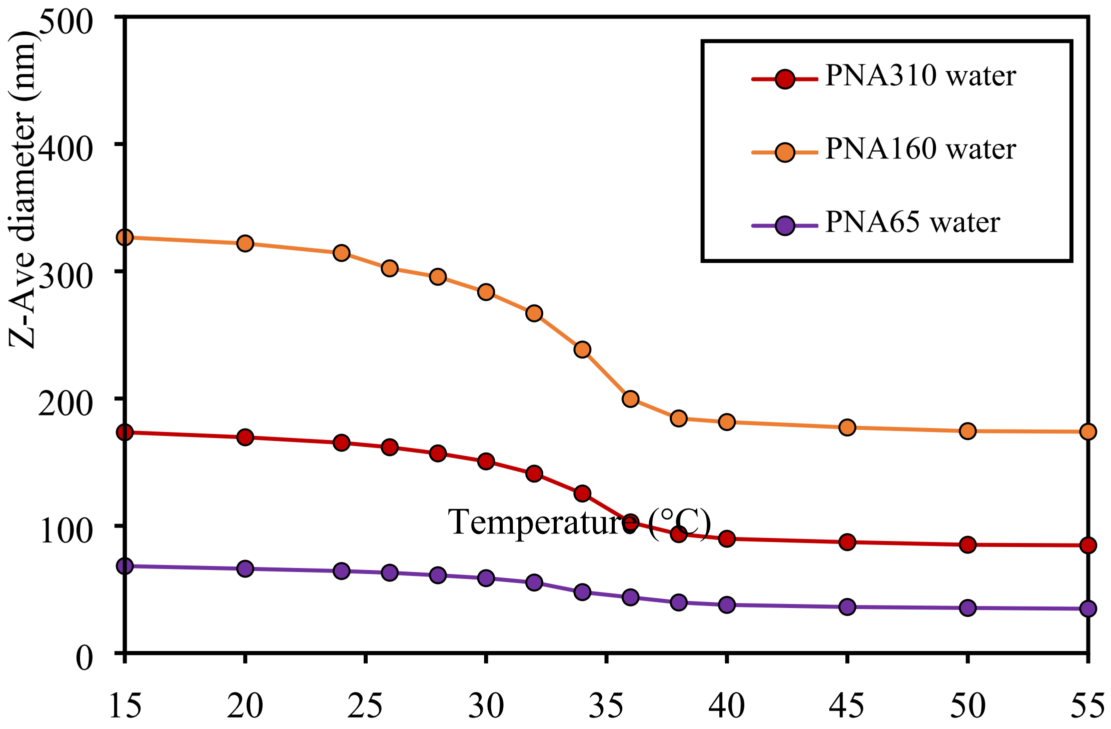

Figure S1. The thermoresponsive behaviour of the PNIPAM nanogels in water as characterised by dynamic light scattering. The particles undergo a deswelling transition at the volume phase transition temperature of 34 °C. This behaviour is exemplified by PNA65, PNA160 and PNA 310.

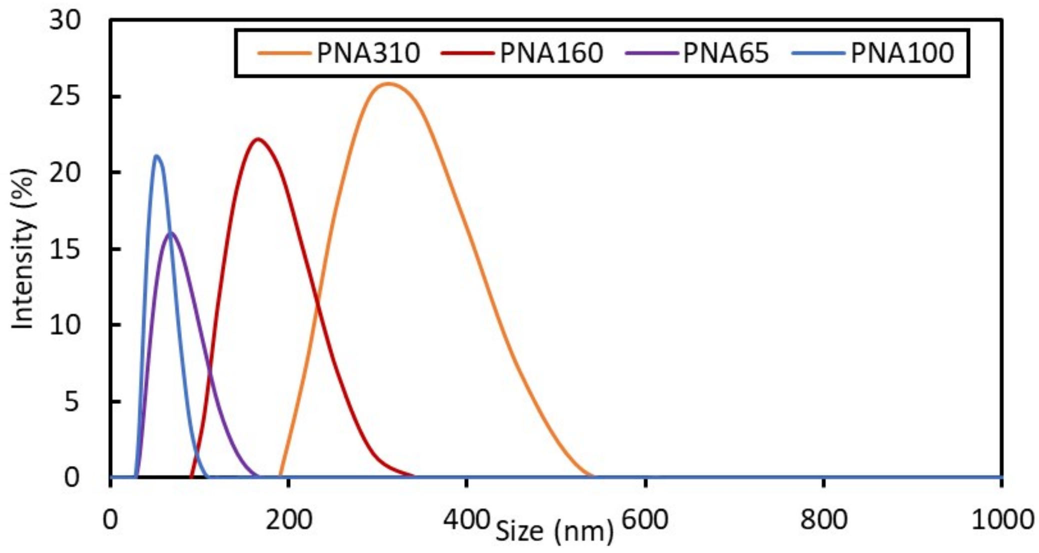

Figure S2. Size distribution analysis by DLS for samples PNA65 (violet solid line), PNA100 (blue solid line), PNA160 (red solid line), PNA310 (orange solid line).

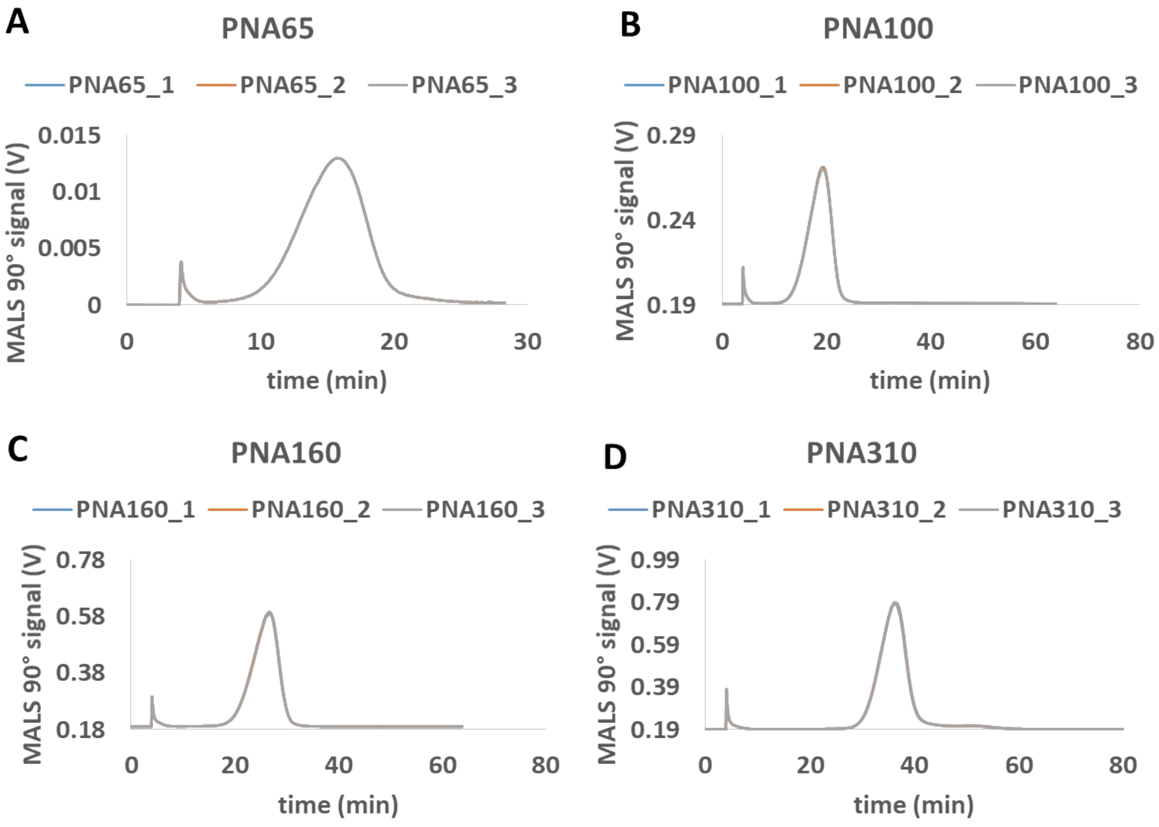

Figure S3. The reproducibility data for three repeat measurements of the different nanogel samples. A) PNA65, B) PNA100 C) PNA160 D) PNA310 using cross-flow 1 mL/min.

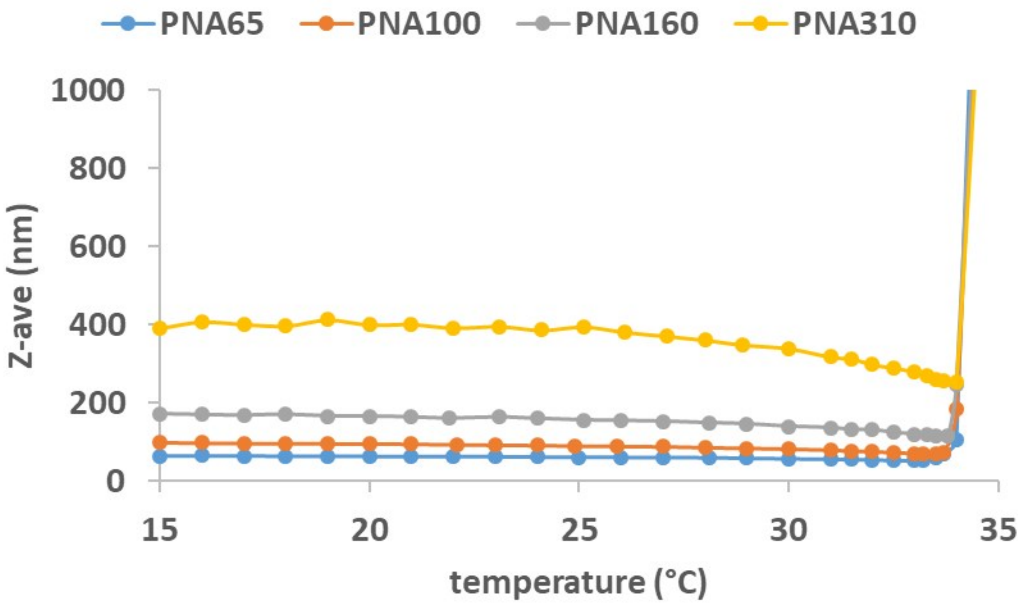

Figure S4. Hydrodynamic diameter of nanogels in 0.1 M $NaNO_3$ measured using DLS at 1 mg ml^{-1} with respect to the nanogel concentration. The sample of the nanogels aggregated at 34 °C and produced then particles sizes that could not be accurately measured by DLS.

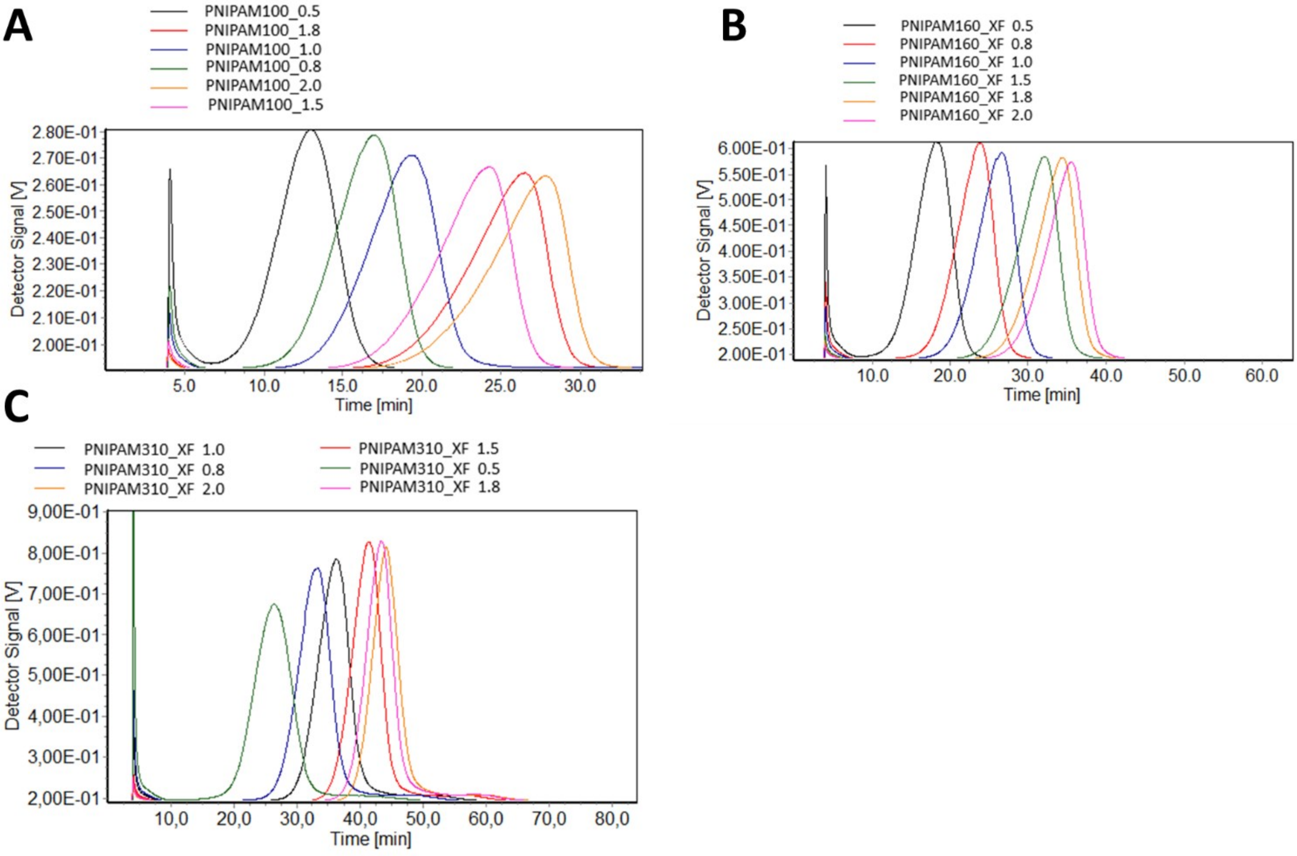

Figure S5. The effect of varying the crossflow on the elution time for the PNA100, PNA160 and PNA310 in $NaNO_3$.

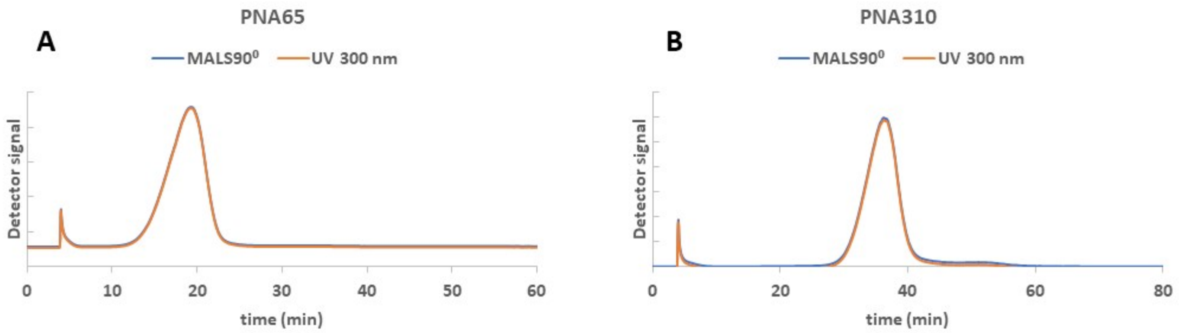

Figure S6. Comparison of the MALS90 signal and UV-vis signal (300 nm) for sample A) PNA65 B) PNA310 using a crossflow of 1 mL/min.

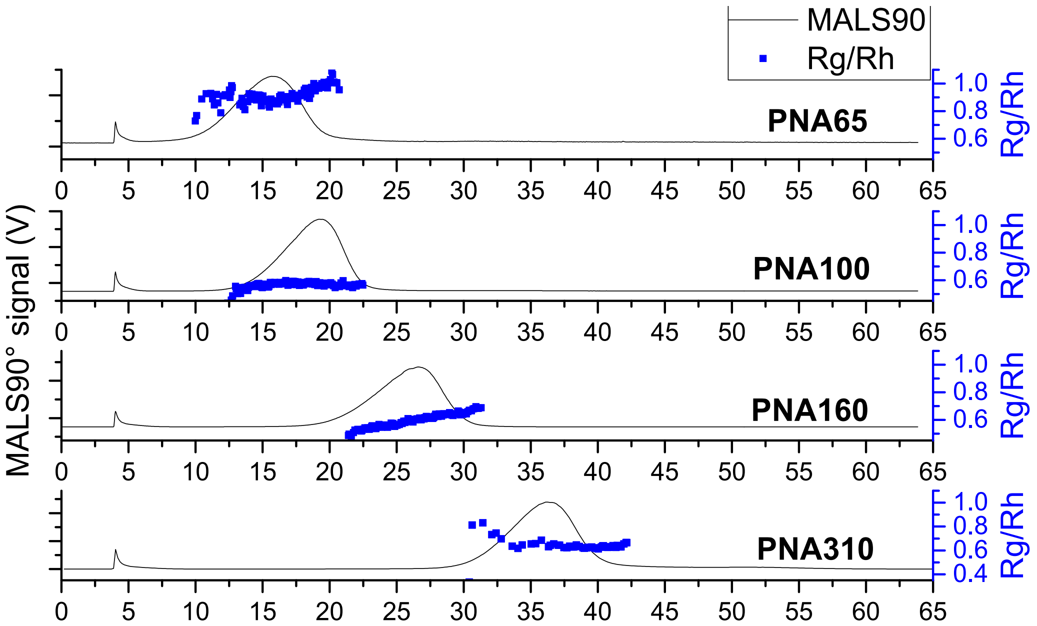

Figure S7. AF4-MALS-DLS analysis of nanogels. Fractograms showing the light 90° scattering detector signal (black line, left hand scale) and shape factor (blue dotted line, right hand scale) obtained from AF4-MALS-DLS measurements for PNA65, PNA100, PNA160 and PNA310.

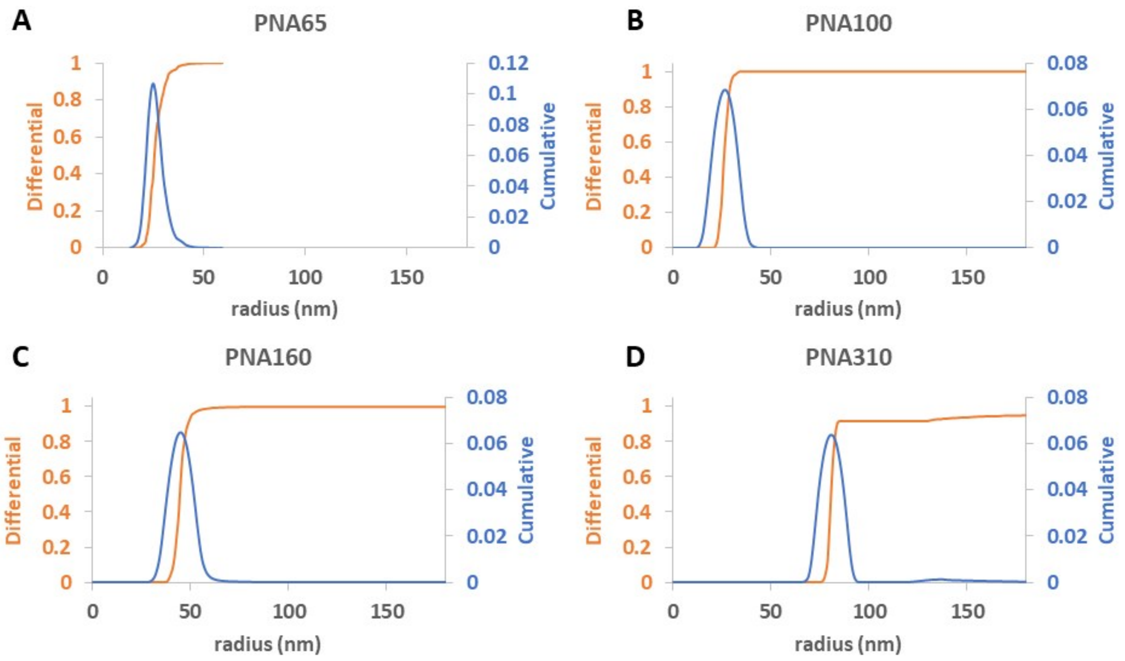

Figure S8. Particle size distributions obtained from AF4-UV-VIS-MALS measurements for the differently sized nanogels A) PNA65, B) PNA100, C) PNA160, D) PNA310.

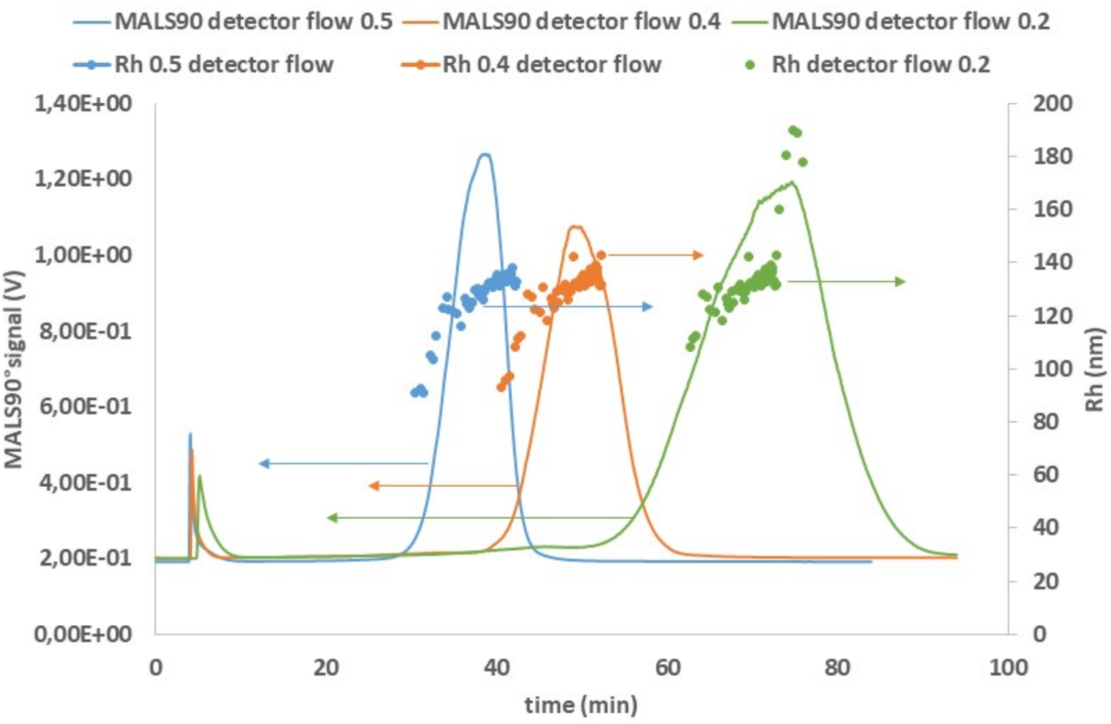

Figure S9. AF4-MALS-DLS analysis of PNIPAM310. Fractograms showing the light 90° scattering detector signal with an initial cross-flow of 1 mL/min and three different detector flows: 0.5 mL/min; 0.4 mL/min and 0.2 mL/min (blue, orange and green line, left hand scale) and R_h (blue, orange and green dotted line, right hand scale) obtained from AF4-MALS-DLS measurements.

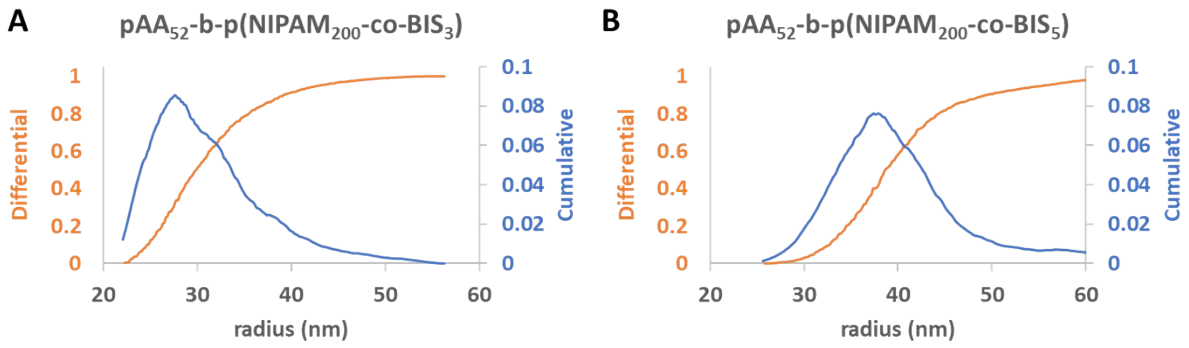

Figure S10. Particle size distributions obtained from AF4-UV-VIS-MALS measurements for the copolymer nanogels prepared by controlled polymerisation A) pAA_{52}-b-p($NIPAM_{200}$-co-BIS_5) and B) pAA_{52}-b-p($NIPAM_{200}$-co-BIS_3).

Synthesis of 2-(hydroxyethylthiocarbonothioylthio)-2-methylpropanoic acid

2-mercaptoethanol (6.1 mL, 87.54 mmol) and potassium phosphate tribasic (19.16 g, 87.54 mmol) were added to acetone (150 mL). Carbon disulfide (11 mL, 0.17 mol) was added to the mixture after 30 min, followed by the addition of 2-bromoisobutyric acid (14.92 g, 87.54 mmol). The mixture was left to stir for 16 h followed by N_2 purging for extra 2 h. Afterwards, the mixture was filtered to remove solids and volatiles were removed under reduced pressure. The product was acidified by addition of hydrochloric acid (1 M, 2 × 100 mL) and the organic phase was extracted with DCM (2 × 100 mL). The organic phase was then washed with a saline solution (100 mL). All the organic phases were collected and dried over Na_2SO_4 and the volatiles removed under reduced pressure. Finally, the crude product was recrystallised from a mixture of hot hexane and ethyl acetate (7:3 v/v ratio) to give a bright yellow solid product (17.2 g, 86 %); m.p. 62-64 °C; δH (400 MHz; $CDCl_3$, 25 °C) (ppm): 3.86 (2H, m, CH_2OH), 3.53 (2H, t, CH_2SC), 1.72 (6H, s, SC(CH_3)$_2$); δC (100 MHz; $CDCl_3$, 25 °C): 25.5 (C(S)SC(CH_3)$_2$), 39.0 (CH_2SC(S)S), 60.8 (CH_2OH), 177.9 (*C*(O)OH), 220.8 (S*C*(S)S); $\bar{\nu}_{max}$ (ATR) cm^{-1}: 3420 (m, R-OH), 1692 (vs, C=O), 1254 (s, S-CH_2-R), 1380 (s, C(CH_3)$_2$), 1160 (s, C=S), 1053 (vs, R-CH_2-OH), 809 (s, S-C-S); $C_7H_{13}O_3S_3$ (240.4); m/z (TOF MS) $[M+H]^+$: 241.1, calcd. for $C_7H_{13}O_3S_3^+$ 241.4.
